# Preoperative Diagnosis of the Bile Duct Traumatic Neuroma by Endoscopic Ultrasound‐guided Tissue Acquisition: A Case Report

**DOI:** 10.1002/deo2.70406

**Published:** 2026-08-20

**Authors:** Kiyohiro Saito, Shunya Oi, Erina Yoshida, Eri Tanaka, Mai Noujima, Noriaki Yamashita, Shuhei Shintani, Atsushi Nishida, Takuji Iwashita

**Affiliations:** ^1^ Department of Gastroenterology Shiga University of Medical Science Hospital Shiga Japan; ^2^ Clinical Laboratory Shiga University of Medical Science Hospital Shiga Japan; ^3^ Division of Diagnostic Pathology Shiga University of Medical Science Hospital Shiga Japan

**Keywords:** amputation neuroma, bile duct lesion, cystic duct stump, EUS‐TA, traumatic neuroma

## Abstract

Traumatic neuroma of the cystic duct stump is a rare benign lesion that may mimic biliary malignancy, making preoperative diagnosis challenging. We report a case of an enhancing lesion at the cystic duct stump after cholecystectomy that was diagnosed by endoscopic ultrasound‐guided tissue acquisition (EUS‐TA). Imaging studies revealed an 8‐mm enhancing nodule at the cystic duct stump, and endoscopic retrograde cholangiopancreatography showed a unilateral filling defect at the corresponding site. Peroral cholangioscopy demonstrated smooth mucosa without irregular vessels, suggesting a submucosal lesion rather than mucosal malignancy. EUS revealed a well‐demarcated, homogeneous hypoechoic mass immediately distal to the origin of the remnant cystic duct. EUS‐TA using a 22‐gauge needle enabled TA from the lesion. Histopathological examination showed proliferation of spindle‐shaped cells with wavy nuclei, and immunohistochemical staining was positive for S‐100 protein, confirming the diagnosis of traumatic neuroma. No procedure‐related adverse events occurred, and unnecessary surgical resection was avoided. EUS‐TA may facilitate preoperative diagnosis of cystic duct stump traumatic neuroma and help guide appropriate management.

**Abbreviations**
CT: computed tomography
ERCP: endoscopic retrograde cholangiopancreatography
EUS: endoscopic ultrasound
EUS‐TA: endoscopic ultrasound‐guided tissue acquisition
MRI: magnetic resonance imaging
POCS: peroral cholangioscopy.

## 1 Introduction

Traumatic neuroma, also known as amputation neuroma, of the bile duct is a reactive hyperplastic lesion characterized by disorganized proliferation of axons and Schwann cells at the site of nerve injury, and it typically occurs after biliary surgery [1]. It is well known that this lesion is difficult to distinguish from cholangiocarcinoma on imaging studies [2]. Therefore, most cases are diagnosed only after surgical resection [3]. Here, we report a case of traumatic neuroma of the bile duct diagnosed by endoscopic ultrasound‐guided tissue acquisition (EUS‐TA), which allowed conservative management, along with a brief review of the literature.

## 2 Case Report

**FIGURE 1:**
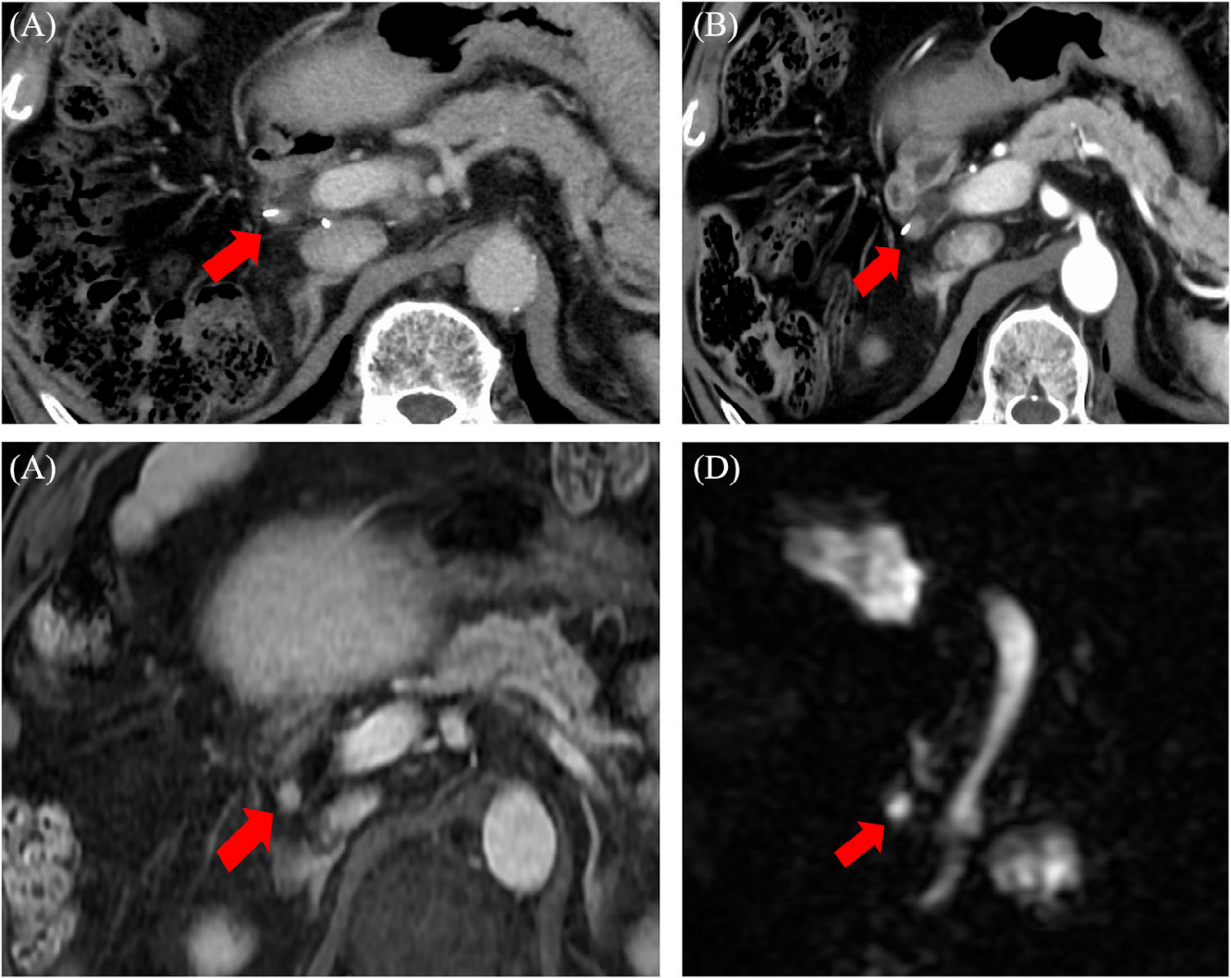
Radiological findings of the lesion. (A) Contrast‐enhanced computed tomography (CT) obtained 4 years before presentation showed a small enhancing nodule at the stump of the remnant cystic duct (red arrow). (B) Arterial‐phase contrast‐enhanced CT demonstrated an 8‐mm enhancing mass at the stump of the remnant cystic duct (red arrow). (C) Arterial‐phase gadolinium ethoxybenzyl diethylenetriamine pentaacetic acid‐enhanced magnetic resonance imaging (EOB‐MRI) showed a well‐defined enhancing mass at the stump of the remnant cystic duct (red arrow). (D) Coronal magnetic resonance cholangiopancreatography (MRCP) demonstrated the remnant cystic duct (red arrow), corresponding to the location of the lesion. No biliary stricture or upstream bile duct dilatation was observed.

**FIGURE 2:**
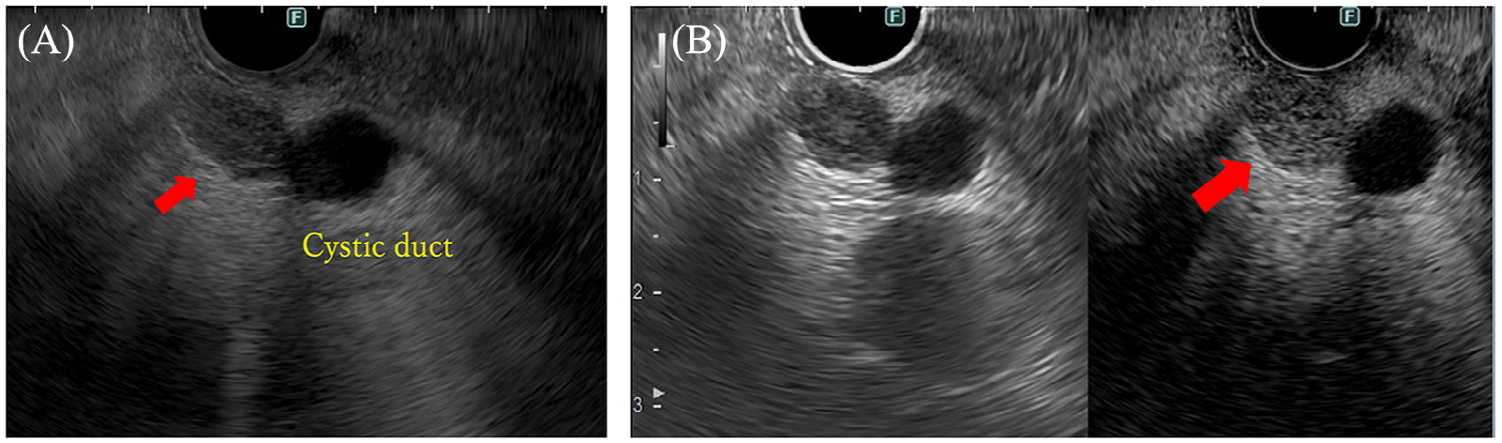
Endoscopic ultrasonographic findings. (A) Endoscopic ultrasound (EUS) demonstrated an 8‐mm well‐defined homogeneous hypoechoic lesion within the remnant cystic duct (red arrow). (B) Contrast‐enhanced EUS using perflubutane (Sonazoid; GE Healthcare, Tokyo, Japan) demonstrated marked enhancement of the lesion at 15 s after contrast injection (red arrow).

**FIGURE 3:**
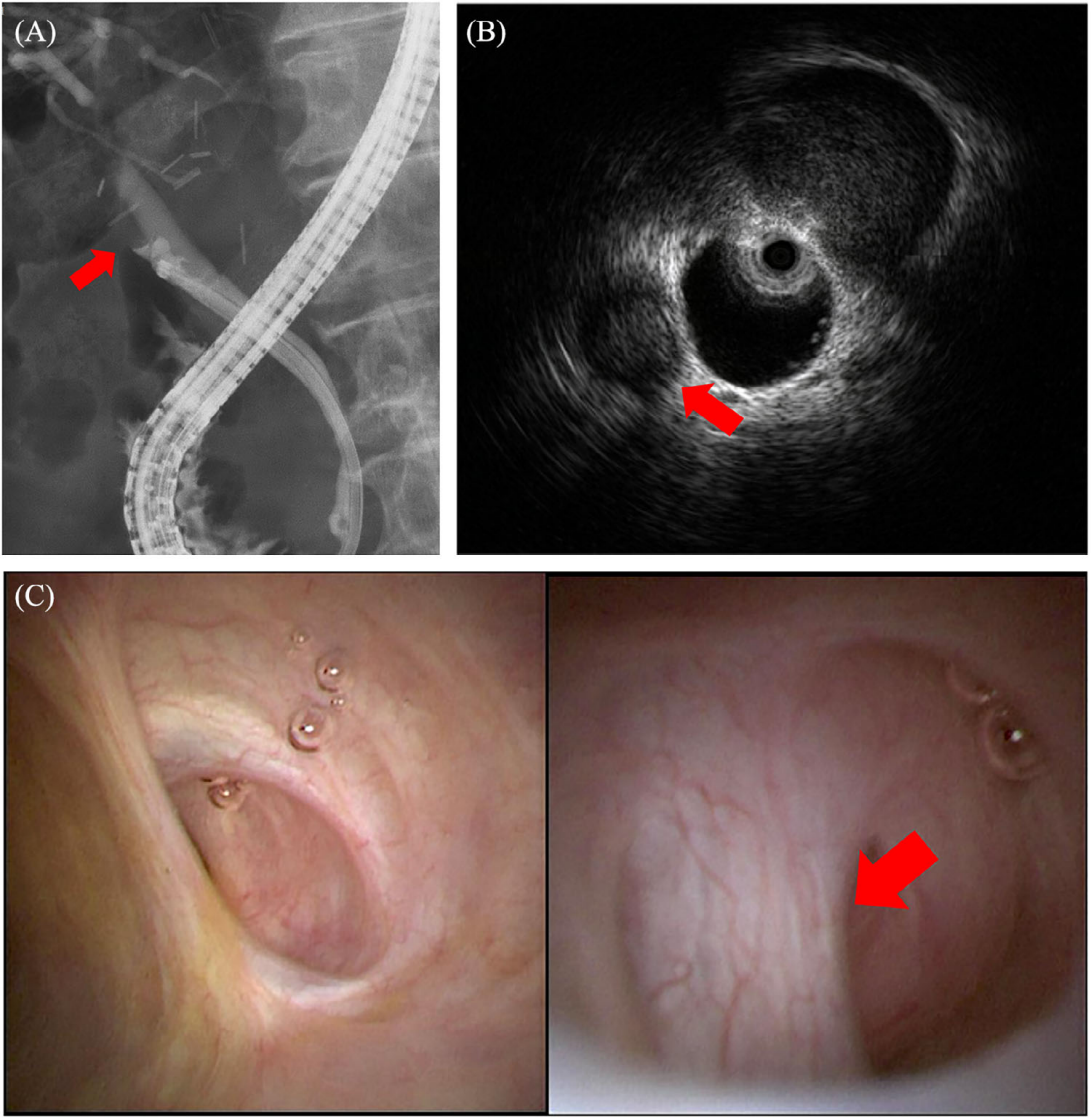
Endoscopic findings. (A) Cholangiography demonstrated an eccentric filling defect at the remnant cystic duct stump (red arrow). (B) Intraductal ultrasonography (IDUS) demonstrated a well‐defined hypoechoic lesion within the remnant cystic duct without disruption of the adjacent bile duct wall (red arrow). (C) Peroral cholangioscopy showed a smooth mucosal surface with a smooth submucosal‐like elevation at the remnant cystic duct stump (red arrow).

**FIGURE 4:**
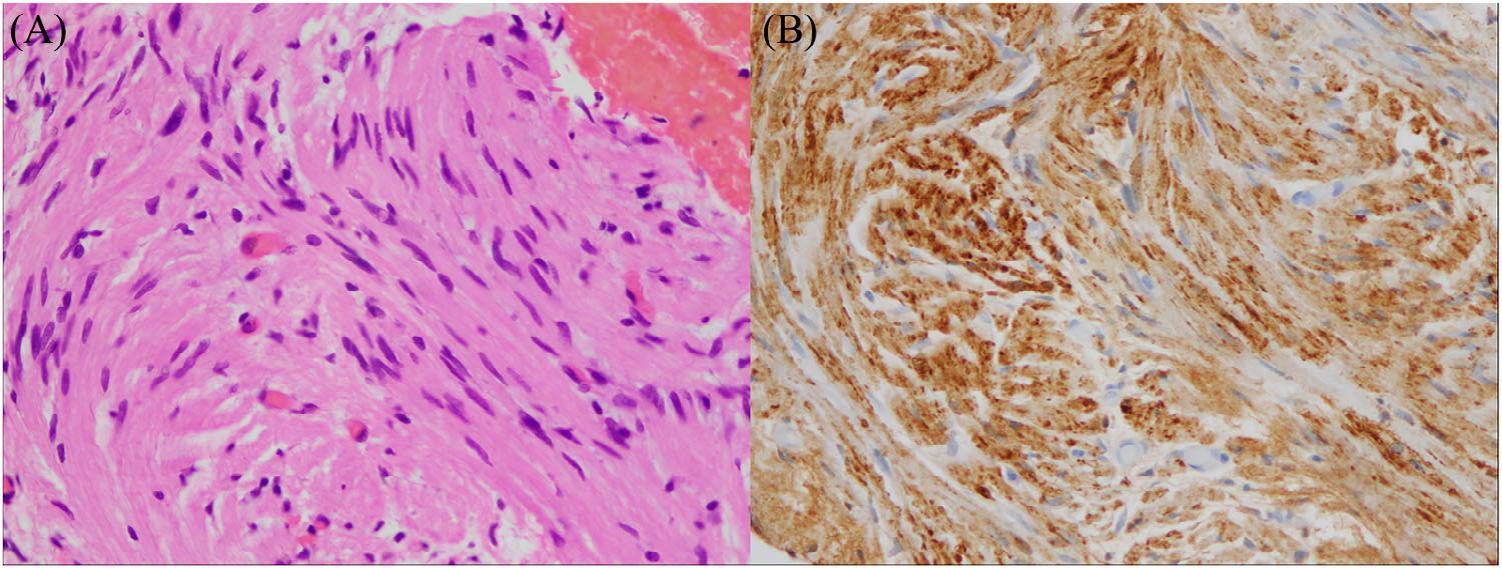
Histopathological findings. (A) Hematoxylin and eosin staining showed proliferation of spindle‐shaped cells with wavy nuclei without nuclear atypia (original magnification ×400). (B) Immunohistochemical staining demonstrated diffuse positivity for S‐100 protein (original magnification ×400).

A 77‐year‐old man had undergone laparoscopic right hepatectomy and cholecystectomy for hepatocellular carcinoma 6 years earlier and had been followed with regular contrast‐enhanced computed tomography (CT). Follow‐up CT showed an 8‐mm nodular lesion at the cystic duct stump, with strong enhancement in the arterial phase and persistent enhancement in the delayed phase, and a gradual increase in size (Figure 1A,B). Surgical clips from the previous cholecystectomy were present adjacent to the lesion. No postoperative complications related to the previous surgery were documented. The patient had no symptoms, and laboratory findings, including serum tumor markers (CEA and CA19‐9), were normal. Gadolinium ethoxybenzyl diethylenetriamine pentaacetic acid‐enhanced magnetic resonance imaging and magnetic resonance cholangiopancreatography showed a well‐defined enhancing nodule at the cystic duct stump, with early enhancement and persistent enhancement in the delayed phase on T1‐weighted imaging (Figure 1C,D). Diffusion‐weighted imaging showed low signal intensity. Endoscopic ultrasound (EUS) showed an 8‐mm well‐defined, homogeneous hypoechoic mass at the cystic duct stump. No wall thickening of the cystic duct was observed. Contrast‐enhanced EUS using perflubutane (Sonazoid; GE Healthcare, Japan) demonstrated marked enhancement of the lesion in the early vascular phase (15 s after contrast injection) (Figure 2). In the retrospective review, the lesion had been visible on CT for 4 years and showed slow growth. Although a benign lesion was suspected, further evaluation was performed. Endoscopic retrograde cholangiopancreatography (ERCP) with peroral cholangioscopy (POCS) was performed. Cholangiography showed a crab claw‐like filling defect at the cystic duct stump (Figure 3A). Intraductal ultrasonography showed a well‐defined, homogeneous hypoechoic lesion without apparent extramural growth (Figure 3B). Peroral cholangioscopy (eyeMAX; Micro‐Tech, China) reached the cystic duct stump and showed a smooth mucosal surface with a smooth submucosal‐like elevation into the lumen, without irregular vessels (Figure 3C). Tissue sampling by cytology or cholangioscopy‐guided biopsy was considered difficult. Therefore, EUS‐TA for the tumor was performed through the duodenal bulb using a 22‐gauge needle (Acquire; Boston Scientific, USA). Two passes were performed using the slow‐pull and dry suction techniques with 10 mL negative pressure [4]. No procedure‐related adverse events occurred. Histopathological examination showed proliferation of spindle cells with wavy nuclei on hematoxylin and eosin staining, which were positive for S‐100 (Figure 4). No Antoni A/B architecture or Verocay bodies were identified. No nuclear atypia, mitotic figures, necrosis, or invasive growth was observed. Based on these findings and the clinical course, the lesion was diagnosed as a traumatic neuroma of the bile duct. The patient was managed conservatively without surgery. Follow‐up CT at 6 months showed no increase in size or new findings.

## 3 Discussion

A variety of lesions can arise at the cystic duct stump, including cholangiocarcinoma, schwannoma, inflammatory pseudotumor, and traumatic neuroma [1, 3]. In the present case, the patient was asymptomatic, and neither laboratory data nor imaging findings strongly suggested malignancy. Although tissue sampling under ERCP was technically difficult, EUS‐TA permitted targeted sampling of the submucosal component and led to a definitive pathological diagnosis of traumatic neuroma. This was clinically significant, as it allowed unnecessary surgical resection to be avoided.

Traumatic neuroma is a reactive hyperplastic lesion characterized by disorganized proliferation of axons and Schwann cells at the site of nerve injury [1]. The incidence of traumatic neuroma of the bile duct has been reported to be 0.23% after biliary surgery in Japan [2]. In contrast, autopsy studies have shown that traumatic neuromas are present in approximately 10%–28% of patients after cholecystectomy [5]. Surgical clips were present adjacent to the lesion in the present case; however, their contribution to the development of the traumatic neuroma could not be determined. Although traumatic neuroma is generally regarded as rare, many cases may remain clinically unrecognized because of the absence of symptoms.

Traumatic neuroma of the bile duct is a benign lesion characterized by slow progression compared with cholangiocarcinoma. However, its imaging features may closely resemble those of cholangiocarcinoma. It can present as an enhancing nodular lesion or biliary stricture and may show focal bile duct wall thickening or intraluminal protrusion [3]. On contrast‐enhanced CT and magnetic resonance imaging (MRI), traumatic neuroma typically appears as an enhancing nodule, whereas ERCP may demonstrate biliary stricture or a filling defect [3]. Therefore, imaging findings are often insufficient to reliably distinguish traumatic neuroma from cholangiocarcinoma [3].

Several findings may support a diagnosis of traumatic neuroma. EUS typically demonstrates a homogeneous hypoechoic mass without evidence of invasion, and diffusion‐weighted MRI often shows low signal intensity [1]. In addition, POCS usually reveals a smooth mucosal surface with a submucosal tumor‐like appearance [6]. Nevertheless, even when these findings are present, differentiation from cholangiocarcinoma remains difficult in some cases, and many patients are ultimately diagnosed with traumatic neuroma only after surgical resection and pathological examination [3]. Because surgical resection for traumatic neuroma may be unnecessarily invasive, particularly in patients without obstructive jaundice, establishing a preoperative pathological diagnosis is important.

For preoperative diagnosis, several reports have described traumatic neuroma diagnosed by cholangioscopy‐guided deep biopsy, also referred to as boring biopsy [6, 7]. However, conventional biopsy may obtain only superficial mucosal tissue and may fail to sample the tumor component; the specimens may show only non‐atypical epithelium or inflammatory cells [3]. This limitation is likely related to the submucosal location of traumatic neuroma, which makes adequate TA difficult. In contrast, EUS‐TA has the advantage of allowing tissue sampling from submucosal or extraluminal lesions via an extramural approach. In the present case, EUS‐TA provided sufficient tissue for pathological evaluation and led to a definitive diagnosis of traumatic neuroma. Although S‐100 positivity is also observed in schwannoma and EUS‐TA has been reported to be useful for the preoperative diagnosis of schwannoma [8], characteristic histological features of schwannoma, including Antoni A/B architecture and Verocay bodies, were absent in the present case. To the best of our knowledge, no previous report has described the preoperative pathological diagnosis of bile duct traumatic neuroma by EUS‐TA.

A meta‐analysis comparing the diagnostic performance of EUS‐TA and ERCP‐based tissue sampling for suspected malignant biliary strictures showed that the sensitivity of EUS‐TA was 75%, which was significantly higher than that of ERCP‐based sampling at 49% [9]. In addition, serious adverse events associated with EUS‐TA are uncommon. Therefore, when traumatic neuroma is included in the differential diagnosis of a biliary stump lesion, EUS‐TA may be useful for obtaining a pathological diagnosis and avoiding unnecessary surgery. However, EUS‐TA for biliary lesions carries potential risks, including peritoneal seeding and bile leakage leading to peritonitis [10]. Thus, the indication for EUS‐TA and the puncture route should be carefully considered.

In this case, EUS‐TA enabled histological diagnosis of traumatic neuroma of the bile duct stump and helped avoid unnecessary surgical resection. Although further accumulation of cases is needed, EUS‐TA may be useful for determining the management of suspected traumatic neuroma.

## Author Contributions

**Kiyohiro Saito** wrote the manuscript. **Kiyohiro Saito**, **Takuji Iwashita**, **Shunya Oi**, **Erina Yoshida**, **Noriaki Yamashita**, and **Shuhei Shintani** performed the endoscopic procedures and managed the patient. All authors reviewed and approved the final manuscript.

## Funding

The authors have nothing to report.

## Conflicts of Interest

Takuji Iwashita is a Deputy Editor‐in‐Chief of DEN Open and was not involved in the peer review process or editorial decision for this manuscript. The other authors declare no conflicts of interest.
